# Ecofriendly colorimetric set-up coupled with mathematical filtration strategy for simultaneous determination of ipratropium and fenoterol in their novel anti-asthmatic metered dose inhaler

**DOI:** 10.1186/s13065-025-01397-2

**Published:** 2025-02-08

**Authors:** Salma N. Ali, Hoda M. Marzouk, Ahmed S. Fayed, Samah S. Saad

**Affiliations:** 1 Pharmaceutical Analytical Chemistry Department, College of Pharmaceutical Sciences and Drug Manufacturing, Misr University for Science & Technology, 6th of October City, Giza, Egypt; 2 Pharmaceutical Analytical Chemistry Department, Faculty of Pharmacy, Cairo University, Kasr Al-Aini Street, Cairo, 11562 Egypt

**Keywords:** Asthma, Colorimetry, COPD, Ipratropium, Fenoterol, Simultaneous equation method, Sustainable analysis

## Abstract

Ipratropium bromide (IPR) and fenoterol hydrobromide (FEN) are well-known medications for treating asthma and chronic obstructive pulmonary disease (COPD). A simple, feasible, efficient, and cost-effective colorimetric assay has been established for determination of the newly introduced co-formulated metered dose inhaler (Atrovent^®^ comp HFA). The developed method is based on the properly optimized reaction of drugs under study with the charge transfer reagent 2,3-dichloro-5,6-dicyano-1,4-benzoquinone (DDQ), resulting in orange-colored complexes measured at 464.3 and 514.0 nm for IPR and FEN, respectively. Smoothly applicable and easily manipulated resolution method, Vierordt’s method, based on straightforward mathematical equations that do not need complicated software was implemented for the simultaneous determination of IPR and FEN. The proposed methodology can be applied in routine analysis enabling fast and economical determination of the combined dosage form without the need for previous separation steps. The validity of the proposed colorimetric method was thoroughly assured as per ICH guidelines with acceptable accuracy and precision. The linearity ranges for IPR and FEN were 5.0–55.0 µg/mL and 10.0–40.0 µg/mL, respectively. Using cutting-edge software metric tools, namely the analytical greenness (AGREE), and complementary green analytical procedure index (ComplexGAPI), the greenness profile of the suggested method was clearly evaluated. The method also conformed well to the recently published blueness (BAGI tool) and whiteness (RGB12 tool) concepts.

**Supplementary Information:**

The online version contains supplementary material available at 10.1186/s13065-025-01397-2.

## Introduction

Globally, asthma and chronic obstructive pulmonary disease (COPD) are instances of chronic lung diseases that contribute to morbidity and mortality worldwide [1]. The quality of life of people with asthma and COPD is significantly impacted [2, 3]. Airway constriction caused by airway inflammation drives the clinical symptoms of both disorders [4]. Inhaling bronchodilators can be beneficial for treating the symptoms of airway constriction in adults and children with bronchial asthma or COPD [5]. That is why the healthcare sector has been working on tracking and alleviating these symptoms through the development of a novel medicine combination of fenoterol hydrobromide (FEN) and ipratropium bromide (IPR) [6, 7].

**Fig. 1:**
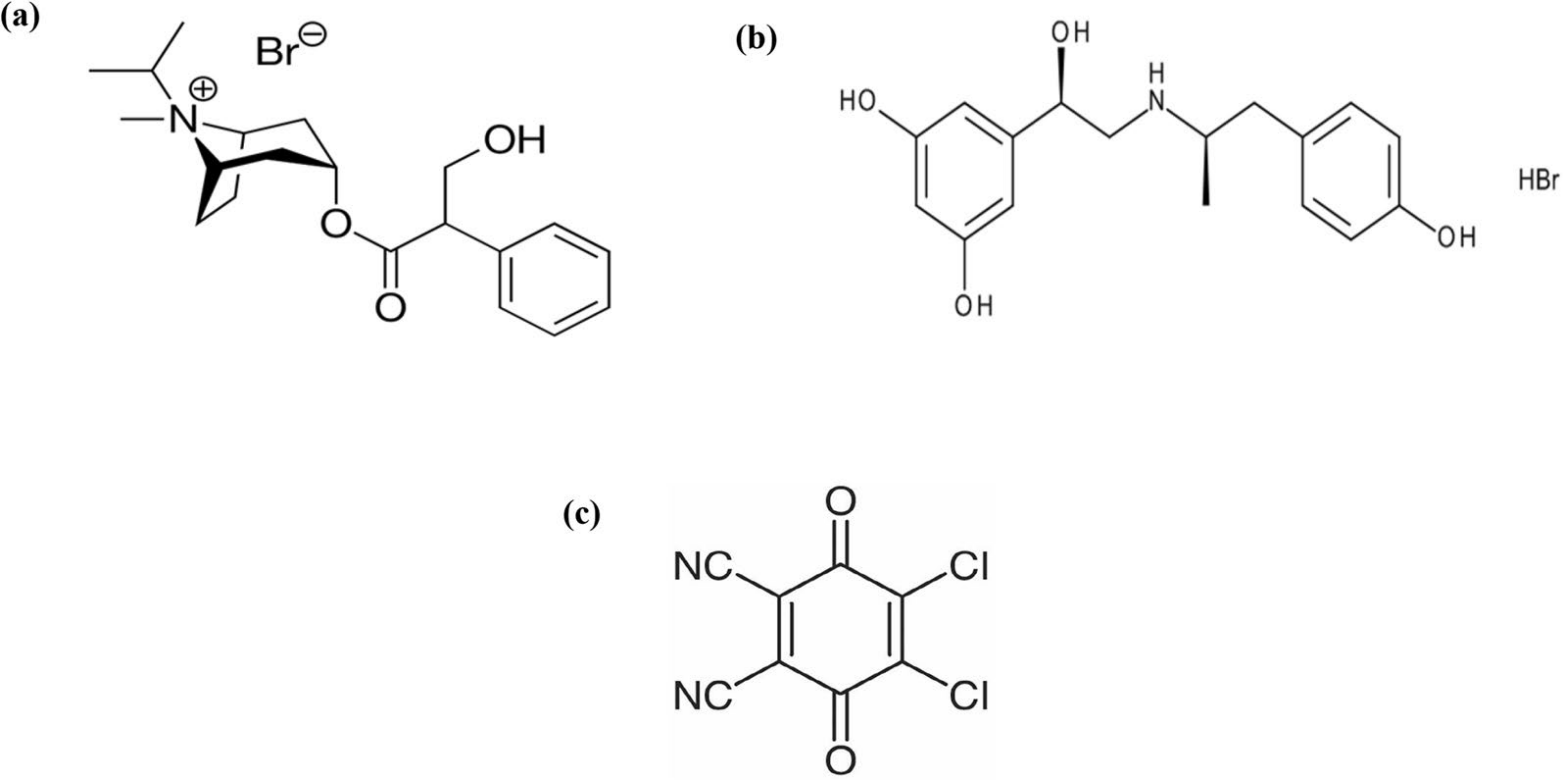
Chemical structures of **a** ipratropium bromide (IPR), **b** fenoterol hydrobromide (FEN) and **c** 2,3-dichloro-5,6-dicyano-p-benzoquinone (DDQ) reagent

Ipratropium bromide (IPR) is an anticholinergic medication (Fig. 1a), licensed by the Food and Drug Administration (FDA) for the treatment of conditions associated with bronchospasm [8]. As a β_2_-adrenergic agonist and inhaled bronchodilator for asthma, fenoterol hydrobromide (FEN), is administered (Fig. 1b) [9]. Recent study results suggest that while IPR and FEN have distinct modes of action, they should be administered in combination rather than separately for the treatment of acute severe asthma and COPD [10]. However, administering each medication at a lower dose in combination may still produce the same clinical result and may lessen the adverse effect [11].

The literature review revealed only two recent analytical approaches for determining IPR and FEN in their co-formulated dosage form: spectrophotometry [12] and chromatography [13]. In addition, a single HPLC method for determining the cited drugs along with other drugs in different single nebulizer solutions was earlier presented [14]. The reported spectrophotometric methods relied on multiple tedious mathematical manipulations and critical measurements. Whereas, the reported chromatographic methods exhibit the limitations of high energy/solvents consumption, using hazardous solvents, and high waste generation, as well as the need for a highly skilled operator and prolonged analysis time.

Spectrophotometric determination of the target drugs in their pharmaceutical formulation is essential but challenging due to the presence of multiple overlapped spectra. The use of inexpensive reagents, low-energy devices, and minimal environmental impact are just a few of the many benefits that spectrophotometric determination offers over other analytical techniques [15, 16]. One of the primary responsibilities and challenges for analytical chemistry researchers is to develop effective derivatization processes for spectrophotometric analysis of weak or non-UV-absorbing substances. It is important to note that no colorimetric sensor is described for the determination of the newly co-formulated mixture of IPR and FEN in the literature. The challenge of analyzing the weak UV-absorbing IPR analyte led us to develop a simple, rapid, cost-effective, and colorimetric charge transfer complexation method for the assay of IPR and FEN using the 2,3-dichloro-5,6-dicyano-p-benzoquinone (DDQ) reagent (Fig. 1c). Colorimetric analysis has multiple applications in pharmaceutical, environmental, and biological analysis [17]. The charge transfer reaction is a well-known colorimetric reaction in which the charge donor analyte reacts with the charge acceptor reagent to produce a stable charge transfer complex with strong absorption in the visible light spectrum [18, 19]. This concept has been previously documented for identifying similar weak UV-absorbing medications in both pharmaceutical dosage forms and their pure form [18, 20].

Accordingly, it seems more challenging to develop a rapid, practical, and enduring colorimetric method for the simultaneous study of IPR and FEN combinations while fully adhering to WAC principles. This study presents simple and intelligent colorimetric strategies to address the highly overlapped and complex spectrum of the studied combination complex. These strategies offer a solution where conventional manipulation methods fall short. The simultaneous equation method (Vierordt’s method) was applied to analyze the native zero-order (°D) absorption spectrum (Window I). The accuracy, precision, and specificity of the proposed approach were rigorously evaluated following the ICH guidelines, and they were found to be successful when applied to evaluating the efficacy of metered-dosage inhalers available on the market. Using reliable and state-of-the-art software metrics such as the analytical greenness (AGREE) and the complementary green analytical procedure index (ComplexGAPI), the effects of the suggested technique on the environment, human health, and safety were assessed. Using the recently developed RGB12 (Red Green Blue 12) and BAGI (Blue Applicability Grade Index) algorithms, the concepts of evaluating “blueness” and “whiteness” were introduced. This emphasized the advantages of the proposed method in terms of analytical performance, sustainability, economy, and practicality.

## Experimental

### Instrument and software

The spectrophotometer (UV-1650; Shimadzu, Tokyo, Japan) equipped with two matching quartz cells with a 1.0 cm path length was used for colorimetric measurements. It is a double-beam UV-Vis spectrophotometer that was aided by UV Probe software (version 2.51). The 400.0–800.0 nm range was scanned at intervals of 0.1 nm. A Jenway pH meter (model 3510) from (Felsted, Essex, UK) was used to adjust the pH.

### Materials and reagents

Global Napi Pharmaceuticals (GNP) Company (Al-Giza, Egypt) kindly provided pure IPR and FEN analytical standards. Their potency was checked and found to be 99.40% ± 0.926 and 99.40% ± 1.062 for IPR and FEN, respectively, according to the BP official methods [21]. Atrovent^®^ comp HFA (Batch No. 104604) is an oral inhalation device with 200 metered doses per metered dose inhaler, manufactured by Boehringer Ingelheim and purchased from a local pharmacy. Each metered dose is labeled to contain 20.0 µg of IPR and 50.0 µg of FEN. Methanol (Fisher Scientific, UK), sodium dihydrogen phosphate (Sigma-Aldrich, Steinheim, Germany), and DDQ (Alfa Aesar Co., Inc.) were all used as analytical-grade chemicals and solvents.

### Stock and working standard solutions

Separate stock standard solutions of IPR and FEN have been prepared at a concentration of 100.0 μg/mL by precisely weighing 10.0 mg of each standard, dissolving and diluting it in 100-mL volumetric flasks with methanol. Various volumes of the appropriate stock standard solution were measured, transferred into a 10-mL volumetric flask, and then diluted with phosphate buffer to prepare additional dilutions. Stock solutions of IPR and FEN remained stable for up to a week when stored at 4 °C and protected from light.

### DDQ reagent and buffer preparation

To prepare the DDQ reagent, precisely 100.0 mg of reagent powder was weighed and transferred into a 100-mL volumetric flask. It was then dissolved and diluted with methanol to achieve the desired concentration of 1.0 mg/mL. A phosphate buffer (10.0 mM) with pH values ranging from 5.0 to 8.0 was prepared, and pH was adjusted using phosphoric acid. The diluent was the prepared buffer.

### Optimization of reaction conditions

#### Effect of dilution solvent

Ethanol, methanol, HEPES buffer, and phosphate buffer were among the solvents used in the reaction of DDQ with the drugs under study to determine the most suitable one for the formation of a charge transfer complex.

#### Effect of pH

To determine the optimal pH for charge transfer complex formation, the reaction of DDQ with the studied drugs was conducted at various pH levels ranging from 5.0 to 8.0.

#### Effect of reagent volume

The impact of reagent volume was studied by adding different volumes of DDQ reagent (100.0 mg%) to a constant concentration (30.0 µg/mL) of the drugs being studied in a buffer as a diluent.

#### Effect of reaction time

The optimal reaction time is determined by monitoring the color intensity growth spectrophotometrically for the DDQ reagent at (25 ± 5 °C) over a specific time interval.

#### Effect of temperature

To study the effect of temperature on the reaction mixture of DDQ with the drugs under study. A series of volumetric flasks under the previously chosen conditions has been placed in water baths with different temperatures (20, 25, 30, 40, and 50 °C) for 5 min.

### Study the reaction stoichiometry

After preparing equimolar solutions of each drug and DDQ, Job’s method of continuous variation [18, 22] was used to calculate the component ratios in each complex. The procedure involved withdrawing different aliquots from each drug and DDQ solution separately and completing the volume with phosphate buffer (pH 7.0). The final volume of the prepared solutions was kept constant, but the molar fraction of each component gradually varied from 0.1 to 0.9. The absorbance of the prepared mixtures was measured at 464.3 nm and 514.0 nm for IPR and FEN, respectively, to determine the drug ratio in each complex.

### General procedure

The calculated volumes of the standard solutions IPR and FEN have been transferred into 10-mL volumetric flasks. Subsequently, 3.0 mL of the reagent solution was added to each sample and mixed thoroughly. The volume was then adjusted using a phosphate buffer (pH 7.0) to reach the desired concentration. The mixture was left for 5.0 min, and the resulting color of the working standard solutions was scanned in the visible range of 400.0–800.0 nm to determine the λ_max_. The maximum absorption values for IPR and FEN are 464.3 nm and 514.0 nm, respectively. The absorbances of resulting solutions of IPR and FEN, in the ranges of 5.0–55.0 μg/mL and 10.0–40.0 μg/mL, respectively, were measured at their respective λ_max_ values. A calibration curve was plotted to determine the linearity and regression equation. The blank was prepared in the same way by replacing the standard solution with a buffer solution.

### Vierordt’s method

If a sample comprises two drugs (x and y) that absorb at each other's maximal absorption (λ_max_), it could be feasible to determine both drugs using the simultaneous equation method (Vierordt's method), as long as specific criteria are met. To meet these criteria, the λ_max_ of the two components must be sufficiently distinct, and there should be no chemical interaction between them. This contradicts the assumption that total absorbance is the sum of the individual absorbances. The absorption of the drugs IPR and FEN at their wavelength maxima established the basis of the simultaneous equation analysis method. 464.3 nm (λ_1_) and 514.0 nm (λ_2_) were the two wavelengths chosen for the simultaneous equation development. The required information was the absorptivity at λ_1_ and λ_2_ of IPR (x) and FEN (y), a_x1_, a_x2_, a_y1_ and a_y2_, respectively. Also, the absorbance of the diluted samples at λ_1_ and λ_2_, A_1_ and A_2_, respectively were required. Let the concentrations of IPR and FEN in the diluted samples be denoted by C_x_ and C_y_, respectively. Based on the knowledge that the mixture's absorbance at λ_1_ and λ_2_ was equal to the sum of the absorbance of x and y separately, two equations were developed. The first equation to determine the IPR concentrations and recovery percentage (% R) was [${C}_{x}=\frac{({A}_{2}{a}_{{y}_{1}}-{A}_{1}{a}_{{y}_{2}})}{{(a}_{{x}_{2}}{a}_{{y}_{1}}-{a}_{{x}_{1}}{a}_{{y}_{2}})}$] and another equation for FEN was [${C}_{y}=\frac{({A}_{1}{a}_{{x}_{2}}-{A}_{2}{a}_{{x}_{1}})}{{(a}_{{x}_{2}}{a}_{{y}_{1}}-{a}_{{x}_{1}}{a}_{{y}_{2}})}$]. Once the equations were established, all that remained was to measure the absorbance of a colored sample solution at two wavelengths and perform simple calculations.

### Method validation

Following ICH guidelines, the developed technique had been effectively validated for linearity, accuracy, precision, linearity, limit of detection (LOD), limit of quantification (LOQ), Robustness, and specificity, proving their suitability for the intended use.

Results validity of the proposed colorimetric method were confirmed via their comparison with those obtained upon applying the corresponding authorized titrimetric and potentiometric titration methods for IPR and FEN, respectively [21]. Statistical analysis was then accomplished using Student *t-*test and F-test.

### Assay of laboratory-prepared mixtures

To assess the specificity and validity of the procedure, seven laboratory mixtures were prepared by adding various ratios of two drugs within their linearity ranges. As previously prescribed for creating synthetic binary mixtures, aliquot volumes from the standard solutions of the drugs under investigation were mixed with 3.0 mL of DDQ reagent in 10-mL volumetric flasks. The solution was then diluted to the appropriate concentration using phosphate buffer (pH 7.0). These mixtures were then scanned spectrophotometrically in the visible range of 400.0–800.0 nm, and the Simultaneous Equation method was used to determine the concentration and percent recovery (%R).

### Application to the pharmaceutical formulation

To achieve the final concentrations of 80 µg/mL of IPR and 200 µg/mL of FEN, precisely 2.0 mL of the metered dose inhaler solution (Atrovent^®^ comp HFA), which is equivalent to 800 µg of IPR and 2000 µg of FEN, were transferred into a 10-mL volumetric flask. The solution was mixed with methanol and then filled up to the mark with the same solvent. For each of the estimated analytes, appropriate dilutions were performed using DDQ reagent and phosphate buffer (pH 7.0) to achieve concentrations within the linear ranges. The previously described procedures were then performed. Using the simultaneous equation method, the concentrations and percent recovery (%R) of each drug were determined. Additionally, the standard addition technique was performed for each drug individually at a claimed concentration of 12 µg/mL. To achieve this, different known amounts of pure IPR and FEN were then added to the dosage form solution. A volume of 3.0 mL of DDQ reagent was added to the mixtures then completed with buffer solution and analyzed as previously described.

## Results and discussion

Spectrophotometry has garnered significant interest in the fields of drug analysis and quality control due to its high throughput, user-friendliness, low solvent and energy consumption per sample analysis [23]. These factors contribute to a clean and environmentally friendly methodology. Spectrophotometric analysis has surpassed conventional chromatographic and electrochemical methods in monitoring the content and potency of available pharmaceutical formulations [24–28]. Colorimetric analysis was utilized for the first time to evaluate the newly co-formulated mixture of IPR and FEN. This work aimed to develop a simple mathematical filter without the need for complicated software manipulation by using an affordable and efficient technique.

### Drug complexes spectra

**Fig. 2:**
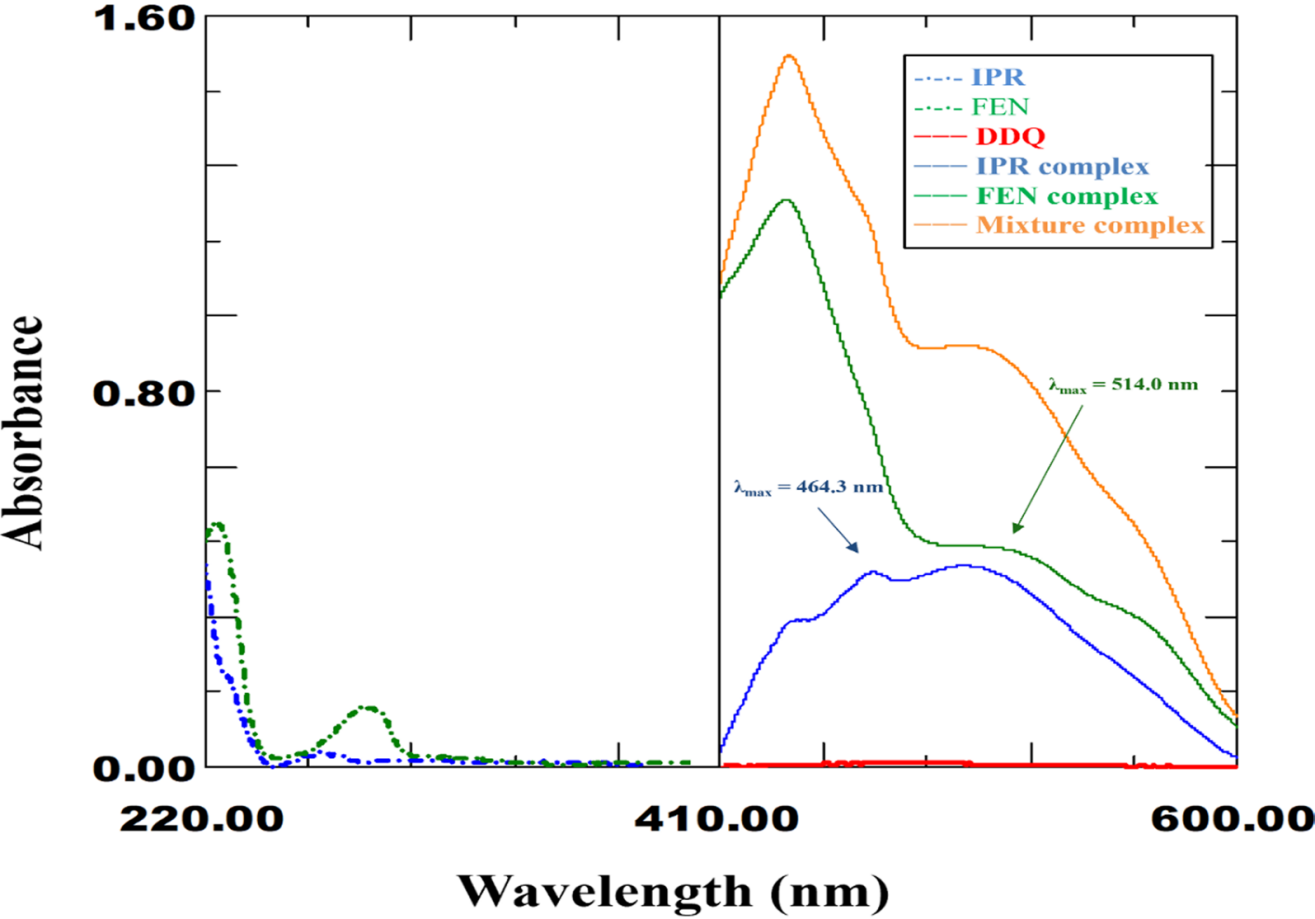
Spectra of IPR (40.0 µg/mL) and FEN (10.0 µg/mL) in UV range (200.0–400.0 nm), along with DDQ (1000.0 µg/mL) and charge transfer complexes of DDQ with IPR (5.0 µg/mL), FENO (20.0 µg/mL) and drug mixture (5.0 µg/mL of IPR and 20.0 µg/mL of FEN) in VIS range (400.0–800.0 nm)

At room temperature (25 ± 5 °C), the DDQ reagent reacts with the examined drugs, IPR and FEN, to form orange-colored complexes that absorb maximally at 464.3 nm and 514.0 nm, respectively. Figure 2 displayed the absorption spectra of DDQ, DDQ-IPR complex, DDQ-FEN complex, and DDQ-Drug mixture complex. Peaks of the formed colored complexes were in the colorimetric range, where the original drug or reagent spectra showed no interference. The colorimetric range referred to the range, where the peaks of the formed complexes were located, without any noticeable interference with the complex formation spectra of the drugs or DDQ reagent. They were helpful in the development of the detection or quantification of portable probes because the colors formed were easily visible to the human eye.

### Method optimization

#### Effect of dilution solvent

The reaction of DDQ with the studied drugs was carried out using different solvents in order to determine the most suitable dilution solvent for charge transfer complex formation. Because of its superior charge transfer complex stability and higher absorbance when compared to the other solvents, phosphate buffer was determined to be the optimum diluent for this reaction.

#### Effect of pH

**Fig. 3:**
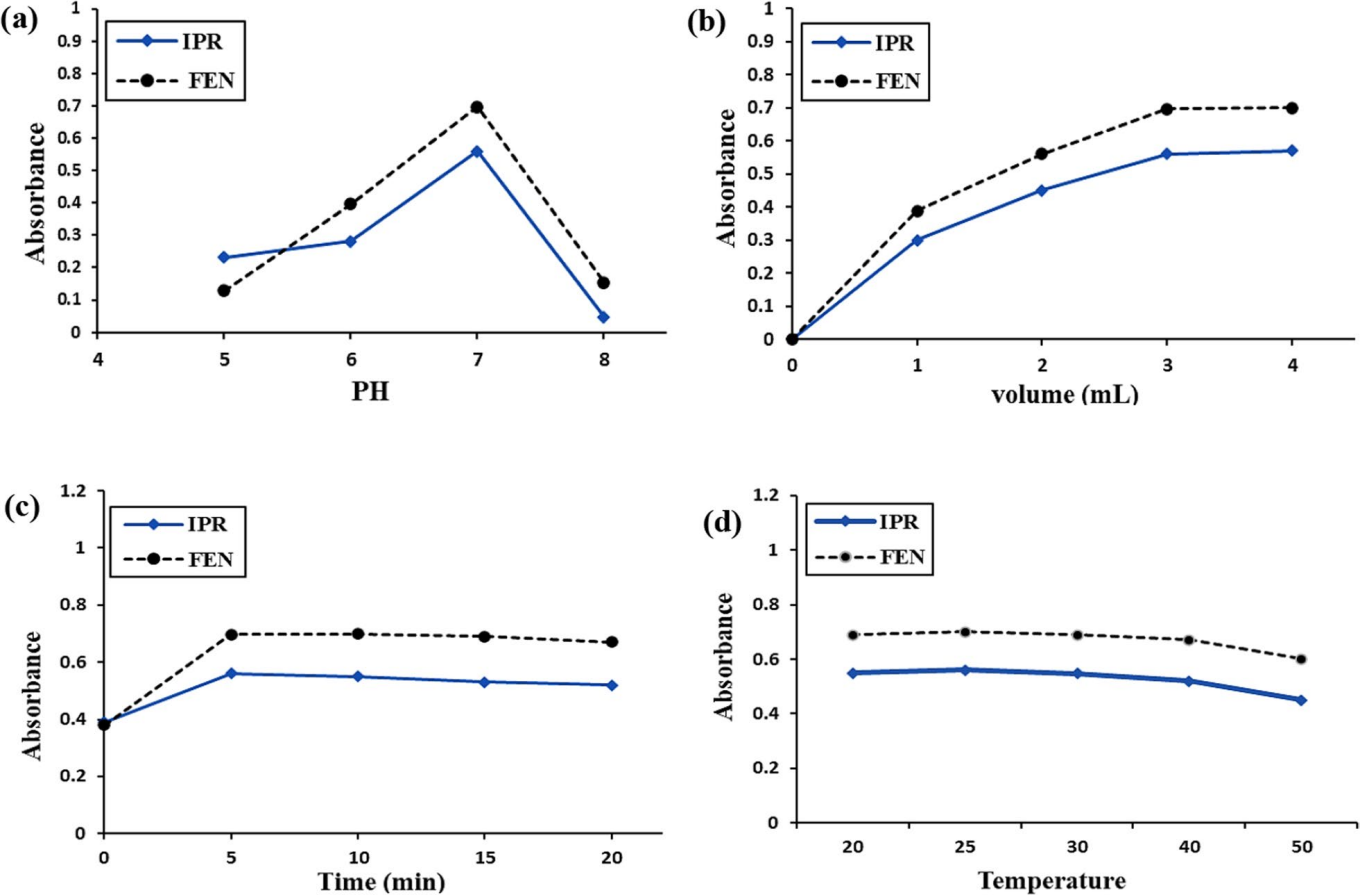
Optimization of various parameters **a** pH of the phosphate buffer, **b** volume of DDQ reagent, **c** standing time, and **d** temperature that affects the absorbance of the charge transfer complex formed between IPR and FEN (30.0 µg/mL) and DDQ reagent

The reaction of DDQ with the studied drugs was carried out at different pH levels ranging from 5.0 to 8.0. Upon using phosphate buffer at pH 7.0 reasonable outcomes for the highest color intensity of the complex formed with IPR and FEN were achieved. Figure 3a illustrates the impact of different pH levels on DDQ charge transfer complexes with the selected drugs.

#### Effect of reagent volume

By increasing the volume of DDQ reagent to 3.0 mL, a gradual increase in the absorbance intensity of the complex formed with IPR and FEN was observed. However, after reaching a certain point, no further increase in intensity was observed (Fig. 3b). It was clear that with each of the drug solutions under study, 3.0 mL of reagent solution was sufficient to produce the highest reproducible color intensity.

#### Effect of reaction time

Although the colored complexes formed immediately, the highest color intensity was observed after 5 min and remained stable for at least 20 min at (25 ± 5 °C). The effect of time on DDQ complexes with the determined drugs is displayed in Fig. 3c.

#### Effect of temperature

The charge transfer complexes demonstrated good stability from 20 °C up to 40 °C. Lower temperatures are therefore preferable for the reactions. A temperature of 25 ± 5 °C was sufficient for quantitative work as shown in Fig. 3d.

### Reaction ratio between studied drugs and DDQ

Job's continuous variation method determined the stoichiometry of the reaction between the cited drugs and the DDQ reagent [18, 22]. Job’s plot was constructed between absorbance (A) versus the respective molar fraction of the cited drugs, Fig. S1. The peak maximum was observed approximately at 0.5 for each drug giving the following drug reagent ratios; ipratropium: DDQ (1:1) and fenoterol: DDQ (1:1) as shown in Fig. S1. The proposed reaction mechanisms for charge transfer between the DDQ reagent and the drugs IPR and FEN were displayed in Fig. S2. In this process, DDQ and the analyzed drugs form a complex in which the electron-deficient benzene moiety of DDQ serves as an electron acceptor, readily acquiring a charge from both IPR and FEN. IPR, characterized by its conjugated electron-rich aromatic ring and ionizable hydroxyl group, efficiently donates electrons and hydrogen to DDQ, thereby facilitating the formation of a colored complex [29, 30]. Furthermore, the tertiary amine structure of FEN features a nitrogen atom with a lone pair of electrons that contributes to the complex formation with DDQ [18].

### Vierordt’s method (Window I)

**Fig. 4:**
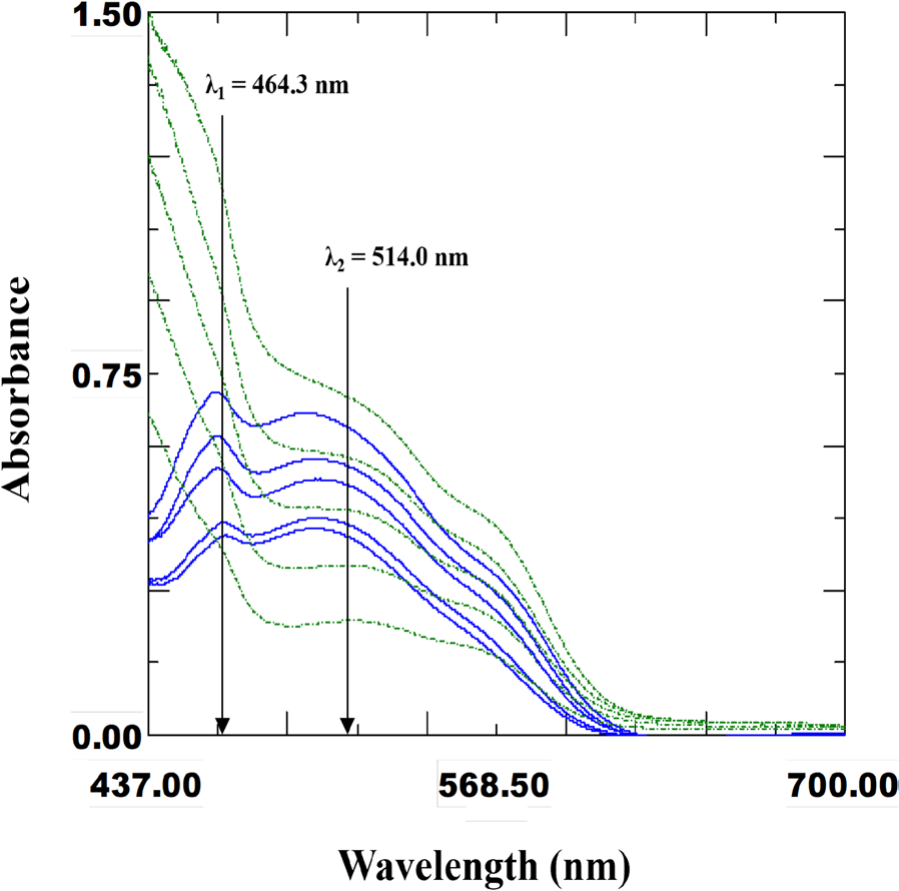
Zero-order absorption spectra of various concentrations of IPR ( ) and FEN ( ) showing the selected wavelengths for Vierordt’s method: 464.3 nm, which is the λ_max_ of IPR and 514.0 nm, which is the λ_max_ of FEN

Vierordt’s method, also known as the simultaneous equation method [31], is one of the simplest ways to resolve overlapped mixture spectra at 464.3 nm and 514.0 nm using only two simple equations. With a precise and inventive mathematical protocol, Vierordt’s method analyzes the °D absorption spectrum in simple steps on the same spectra without the need for a divisor or additional derivatization steps. Therefore, Vierordt’s method is classified as a Window I spectrophotometric platform and a progressive resolution technique. As a result, it can be applied in routine analysis because it enables the straightforward, quick, and direct determination of the combined dosage form without the need for prior separation [32, 33]. Figure 4 displayed the overlapped spectra for both drugs. This method used two wavelengths: λ_1_ = 464.3 nm, which is the λ_max_ of IPR with a significant absorbance of FEN, and λ_2_ = 514.0 nm, which is the λ_max_ of FEN with a significant absorbance of IPR (Fig. 4). At the selected wavelengths, the absorbance was measured, and the absorptivity values for IPR and FEN were calculated. The absorptivity coefficient values calculated for IPR are 0.006 (a_x1_) and 0.0044 (a_x2_), and for FEN are 0.0383 (a_y1_) and 0.0225 (a_y2_). These values were an average of six estimations. Two equations were derived from the simple fact that at λ_1_ and λ_2_, the absorbances of the drug mixture were the sum of the individual absorbances of IPR and FEN. At 464.3 nm, the mixture’s absorbance was equal to

1 $$\left[ {{\text{A}}_{{1}} = {\text{a}}_{{{\text{x1}}}} {\text{c}}_{{\text{x}}} + {\text{a}}_{{{\text{y1}}}} {\text{c}}_{{\text{y}}} } \right],$$

and the absorbance of the mixture at 514.0 nm was

2 $$\left[ {{\text{A}}_{{2}} = {\text{a}}_{{{\text{x2}}}} {\text{c}}_{{\text{x}}} + {\text{a}}_{{{\text{y2}}}} {\text{c}}_{{\text{y}}} } \right]$$

when the measurements were done in 1 cm cells, where b = 1. Then, by arrangement, the last equations would yield the following formulas [${C}_{x}=\frac{{A}_{1}-{a}_{{y}_{1}}{c}_{y}}{{a}_{{x}_{1}}}$] and [${C}_{y}=\frac{{A}_{2}-{a}_{{x}_{2}}{c}_{x}}{{a}_{{y}_{2}}}$], respectively. Substituting for C_x_ in Eq. (2) and C_y_ in Eq. (1), and rearranging gave the following new equations for determining each drug separately in the drug mixture: Eq. (3) for the determination of the IPR concentrations was

3 $$\left[ {C_{x} = \frac{{\left( {A_{2} a_{{y_{1} }} - A_{1} a_{{y_{2} }} } \right)}}{{(a_{{x_{2} }} a_{{y_{1} }} - a_{{x_{1} }} a_{{y_{2} }} )}}} \right]$$

and Eq. (4) for FEN was

4 $$\left[ {C_{y} = \frac{{\left( {A_{1} a_{{x_{2} }} - A_{2} a_{{x_{1} }} } \right)}}{{(a_{{x_{2} }} a_{{y_{1} }} - a_{{x_{1} }} a_{{y_{2} }} )}}} \right].$$

By substituting all the previously calculated absorptivity coefficient values (a_x1_, a_x2_, a_y1_, and a_y2_), the final simplified forms of Eqs. (3) and (4) were obtained. Then Eqs. (3) and (4) could be easily solved by substituting the absorbance values (A_1_ and A_2_) of the drug mixture at 464.3 nm (λ_1_) and 514.0 nm (λ_2_) to determine the IPR and FEN.

### Method validation

The validation criteria of the colorimetric methods were assessed following the ICH guidelines for validating analytical procedures [34].

Table S1 provides a statistical comparison of the results obtained by the suggested method with those obtained using the official ones for analysis of IPR and FEN. The theoretical tabular Student-t and F values were contrasted with the computed ones. Results indicated that the accuracy and precision of the developed and official methods did not differ significantly**.**

#### Linearity and calibration ranges

As previously mentioned, the drugs under investigation were serially diluted and prepared for colorimetric analysis. The calibration curves were constructed to represent the relationship between absorbance (A) and the corresponding concentration in the ranges of 5.0 to 55.0 and 10.0 to 40.0 µg/mL of IPR and FEN, respectively, the regression equations were then obtained. The molar absorptivity values were calculated and found to be 2474.2 L mol^−1^ cm^−1^ and 8645.8 L mol^−1^ cm^−1^ for IPR and FEN in order. Table 1 summarized the parameters of the regression equation assay.

**Table 1:** Method validation parameters and regression for the determination of a binary mixture of ipratropium and fenoterol by the proposed colorimetric method

Parameter	Colorimetric method	
	IPR	FEN
Wavelength (nm)	464.3	514.0
Linearity range (µg/mL)	5.0–55.0	10.0–40.0
Regression equation parameters		
Slope (b)^a^	0.006	0.0225
SE of the slope	0.00002	0.0002
Intercept (a)^a^	0.3814	0.0151
SE of the intercept	0.0006	0.0053
Correlation coefficient (r)	1.0000	0.9998
Accuracy (mean ± %RSD)^b^	100.28 ± 1.83	100.29 ± 1.70
Precision (± %RSD)		
Repeatability^c^	0.914	1.057
Intermediate precision^d^	1.234	1.794
Specificity (mean ± %RSD)	100.28 ± 1.387	99.78 ± 1.337
LOD^e^	0.335	0.604
LOQ^e^	1.014	1.830
Robustness (%RSD)		
Scanning wavelength (± 2 nm)	1.801	1.790
pH (± 0.1)	0.464	1.051

^a^Regression equation for colorimetric method: A = a + bc, where ‘A’ is the average absorbance, c’ is the concentration (μg/mL), ‘a’ is the intercept and ‘b’ is the slope

^b^Accuracy [average of five different concentrations of three replicates each (n = 15)]

^c^Intra-day precision [average of three different concentrations of three replicates each (n = 9) within the same day]

^d^Inter-day precision [average of three different concentrations of three replicates each (n = 9) repeated on three successive days]

^e^LOD and LOQ are calculated according to ICH, 3.3 × SD of the residuals/slope and 10 × SD of the residuals/slope, respectively

#### Limits of detection and quantification

Limit of detection (LOD) and limit of quantification (LOQ) values for IPR and FEN were calculated using the standard deviation of the residuals and the slope of their respective calibration curves (Table 1). The obtained LOD and LOQ values indicated good sensitivity of the proposed method.

#### Accuracy and precision

To assess the accuracy of the reported procedure, five different pure samples in triplicates within the known linearity range of IPR (14.0, 20.0, 32.0, 42.0, and 50.0 µg/mL) and FEN (16.0, 18.0, 23.0, 26.0 and 38.0 µg/mL) were examined using optimized colorimetric conditions. The appropriate regression equation was used to calculate the concentrations of each drug. Achieving acceptable mean percentage recoveries (%R) validated the accuracy of the suggested method (Table 1). Intra- and inter-day precisions were established by repeating the analysis of three different chosen concentrations of IPR (10.0, 30.0, and 40.0 µg/mL) and FEN (20.0, 25.0 and 30.0 µg/mL) three times on the same day and three subsequent days, respectively. Table 1 displayed the results of the calculation of the relative standard deviation (RSD%).

#### Robustness

The robustness of the proposed method was demonstrated by a mean change in the scanning wavelength of ± 2 nm and buffer pH of ± 0.1. All the outcomes were within the permissible range. Because the validation parameters were within an acceptable range and the pooled relative standard deviation (RSD%) was less than 2%, the procedure proved to be robust (Table 1).

#### Specificity

The feasibility of quantifying the investigated drugs in binary mixtures with varying composition ratios simultaneously was evaluated by preparing multiple laboratory-prepared mixtures with different drug concentrations. Table 2 showed the analysis results of acceptable percent recoveries and (%RSD) values to provide insight into the specificity of the methods presented.

**Table 2:** Determination of ipratropium and fenoterol in laboratory-prepared mixtures by the proposed colorimetric method

Mixture No	Claimed concentration (µg/mL)		Recovery %	
			Vierordt’s method	
	IPR	FEN	IPR	FEN
1	10.0	10.0	98.90	99.70
2	10.0	20.0	98.30	101.60
3*	5.0	12.5	100.62	98.40
4*	10.0	25.0	102.00	99.21
5*	15.0	37.5	101.33	98.66
6	20.0	10.0	99.50	102.00
7	55.0	40.0	101.27	98.92
Mean ± RSD%			100.28 ± 1.387	99.78 ± 1.337

^*^ Different laboratory-prepared mixtures prepared at the ratio of dosage form

### Analysis of the pharmaceutical formulation (Atrovent^®^ comp HFA)

The proposed colorimetric method was effectively applied to analyze an Atrovent^®^ comp HFA metered dose inhaler, confirming the absence of any excipient interference. IPR and FEN are selectively determined in their oral inhalation dosage form. Additionally, a standard addition technique was used to ensure the validity of the proposed methods, as shown in Table 3. The developed colorimetric method has the merits of straightforward procedure, economic cost, simple operation, and available instrumentation, and it does not require an experienced person to handle it or complicated software manipulation.

**Table 3:** Results achieved by applying the proposed colorimetric method for the quantitative estimation of ipratropium and fenoterol in their pharmaceutical formulation and application of standard addition technique

Pharmaceutical formulation	Colorimetric method				
	Drug (µg/mL)	%Found ± SD^*^	Standard Addition Technique		
			Claimed (µg/mL)	Pure added (µg/mL)	%Recovery of the pure added^*^
Atrovent^®^ comp HFA inhaler solution (Each metered dose is labeled to contain 20.0 µg IPR and 50.0 µg FEN) (BN. 104604)	IPR (8)	98.00 ± 1.20	12	10.0	98.88
				12.0	101.25
				24.0	100.90
				Mean ± SD	100.35 ± 1.278
	FEN (20)	98.70 ± 0.93	12	10.0	100.67
				12.0	100.00
				24.0	99.86
				Mean ± SD	100.18 ± 0.431

^*^ Average of five determinations

### Greenness profile

To reduce the detrimental impact that analytical techniques have on the environment and public health, the term “Green Analytical Chemistry” (GAC) was developed in 2000 [35]. Achieving the best possible results while simultaneously reducing the environmental risks associated with analysis methods has become imperative. One of the most critical steps in method development is selecting solvents and reagents to prevent their potential negative impact on both the environment and human health [36, 37]. The goal was to create a safe and more environmentally friendly method while also aligning with the global movement towards sustainable chemistry. This study also aimed to demonstrate that it is possible to utilize green analytical techniques without compromising analysis parameters. Additionally, the tools listed below were used to assess and confirm the level of greenness for the proposed methods, namely: the Analytical Greenness metric (AGREE) and the Complementary Green Analytical Procedure Index (Complex GAPI).

#### Analytical greenness metric (AGREE)

**Fig. 5:**
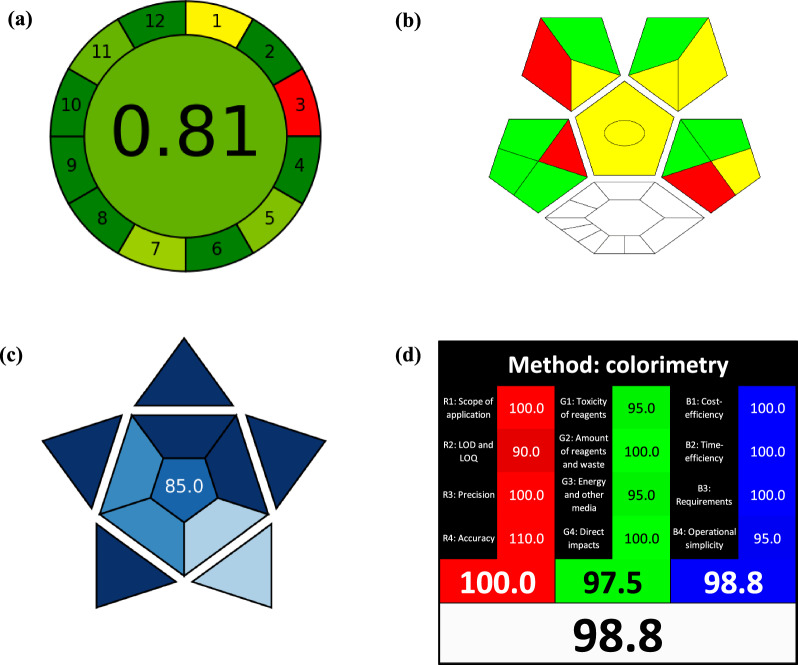
Greenness, blueness, and whiteness assessment of the proposed colorimetric method via **a** AGREE, **b** ComplexGAPI, **c** BAGI, and **d** RGB12 metric tools

It is a cutting-edge tool for assessing greenness [38]. The ultimate AGREE score is a fraction of one, ranging from zero to one. It is an automatically generated pictogram with twelve sections. From deep green (1) to deep red (0), each section has a different color. The final score is located in the center of the circular pictogram. When establishing the AGREE tool, fundamental concepts like inclusivity, simplicity, and flexibility in input and output clarity were taken into consideration [39]. Figure 5 presents AGREE pictograms with only one red zone, which corresponds to off-line sampling. The overall score displayed in the center of the pictogram is 0.81, indicating the highest ecological compatibility and lowest impacts of the proposed methods.

#### Complex green analytical procedure index

This novel and simple tool enhances the original GAPI metric [40]. The ComplexGAPI metric expands five pentagrams of the GAPI tool by a hexagonal field at the bottom. How “green” the pre-analysis processes are is reflected in this field. ComplexGAPI covers every stage of the pre-analysis and analysis process. The improved tool employs a color scale with two or three assessment levels for each step, like the GAPI tool. The generated pictogram can be used to evaluate and quantify the low, medium, and high environmental impact for each step, from green to yellow to red. Different fields represent all of the parts of the processes and analytical protocol that are described. When certain criteria are satisfied, these fields are filled with green [41]. By examining the Complex GAPI pictogram in Fig. 5, it was evident that the green and yellow colors predominate in the pictogram, with fewer red-shaded sections, indicating the prevalence of green analytical methods.

#### The blue applicability grade index (BAGI)

The blue applicability grade index (blueness) is a simple, fast, and recently developed tool. BAGI is developed for the evaluation of the practicality of any analytical method and helps identify the applicability strengths and weaknesses of each method [42]. The blue color is inspired by the RGB model, and the proposed index can be regarded as a complement to the current green metrics instruments. In addition, it adheres to the ideas of environmental sustainability. The primary ten attributes are taken into consideration by the BAGI metric tool when assessing the applicability of an analytical method. Attributes 1–3 relate to the analytical determination step, the sample preparation step is represented by attributes 4 and 5, and both steps are represented by attributes 6–10. The BAGI metric tool yields two different sets of results: an asteroid pictogram as a graphical representation and a numerical score at the center of the pictogram. The asteroid pictogram, which is made up of different blue hues to represent varying degrees of compliance (dark blue for high, blue for moderate, light blue for low, and white for non-compliance), four discrete scores of equal weights are used in the assessment (10, 7.5, 5.0, and 2.5 points). Each score corresponds to a different hue, respectively [42, 43]. Significantly, the pictogram’s level of blueness varied depending on the approach, yielding a score of (85), as shown in Fig. 5.

#### White analytical chemistry (WAC)

The White Analytical Chemistry method (Whiteness) is a novel approach that could be used as an indicator for evaluating sustainable and efficient analytical methods. Paweł Nowak updated the WAC in 2021 [44]. The primary colors red for the method efficacy, green for environmental impact aspects, and blue for practical and economic considerations were used to develop the name of the method. Together, these colors combine to produce the white color of the method. A perfect analytical technique is described as “white” in the WAC concept, denoting a high saturation of each primary color. The WAC tool is also referred to as RGB12 depending on the number of rules involved. With meticulous consideration, the RGB12 tool has been developed to assess methods depending on the 12 WAC principles. By evaluating the “whiteness” of a method, this tool provides some insight into its sustainability. Although the RGB12 Algorithm is not a direct indicator of sustainability, it is a valuable tool because it offers a thorough assessment that considers a wide range of factors [43, 45]. The recommended colorimetric method was investigated using the RGB12 tool and the evaluation results of this method are displayed in Fig. 5. The overall whiteness score is 98.8%. It was determined that the prescribed colorimetric method fully adheres to the WAC principles.

## Conclusion

Atrovent^®^ comp HFA is a newly launched co-formulated inhaler containing IPR and FEN. It is used for the treatment of COPD and asthma. Recently, analytical laboratories and instrumental corporations have embraced the concept of sustainable development. The study successfully achieved the development of a deviceful and user-friendly analytical methodology for the simultaneous measurement of IPR and FEN in their new metered dose inhaler. Due to its superior qualities, such as great solvent sustainability, lower solvent, and energy consumption, independence from highly qualified analysts, and cost- and time-effectiveness, colorimetric analysis was chosen over classical chromatographic methods. The work introduced a smooth and direct colorimetric approach to addressing these challenges. This was achieved by applying a simple mathematical filter to the target drug, utilizing one or more numerical factors, such as the absorptivity factors in Vierordt’s method. To ensure the effectiveness of the metered dose inhaler, the proposed approach was successfully implemented. The study also successfully assessed the sustainability of the proposed method using the AGREE tool and the highly developed ComplexGAPI pictograms, highlighting their superiority in this aspect. Finally, a comprehensive evaluation of the proposed approach using the BAGI and WAC tools was provided, emphasizing the highest level of compliance with the GAC standards.

## Supplementary Information

Supplementary Material 1

## Data Availability

All data generated or analysed during this study is provided within the manuscript or supplementary information files.
