# Supplementary material for: Ecofriendly colorimetric set-up coupled with mathematical filtration strategy for simultaneous determination of ipratropium and fenoterol in their novel anti-asthmatic metered dose inhaler
Source: BMC Chem. 2025 Feb 8;19(1):34. doi: 10.1186/s13065-025-01397-2 (PMC11806664; doi:10.1186/s13065-025-01397-2)
Supplement: Supplementary file 1 — Supplementary Material 1 [file 13065_2025_1397_MOESM1_ESM.docx]

**Supporting Information**

**For**

**Ecofriendly colorimetric set-up coupled with mathematical filtration strategy for simultaneous determination of ipratropium and fenoterol in their novel anti-asthmatic metered dose inhaler**

Salma N. Ali^1^, Hoda M. Marzouk^2^*, Ahmed S. Fayed^2^, Samah S. Saad^1^

*^1^ Pharmaceutical Analytical Chemistry Department, College of Pharmaceutical Sciences and Drug Manufacturing, Misr University for Science & Technology, 6^th^ of October City, Giza, Egypt.*

*^2^ Pharmaceutical Analytical Chemistry Department, Faculty of Pharmacy, Cairo University, Kasr Al-Aini Street, 11562, Cairo, Egypt*

*** Corresponding author:**

Hoda M. Marzouk, Ph.D.

Assistant Professor of Pharmaceutical Analytical Chemistry

Faculty of Pharmacy, Cairo University, Egypt.

**E-mail address:** [hodaallah.marzouk@pharma.cu.edu.eg](mailto:hodaallah.marzouk@pharma.cu.edu.eg)


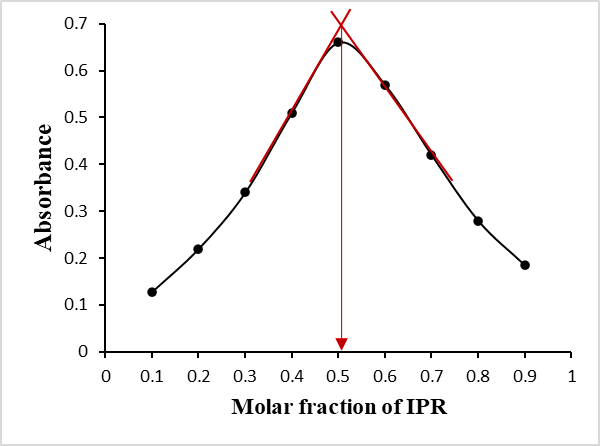


**(a)**


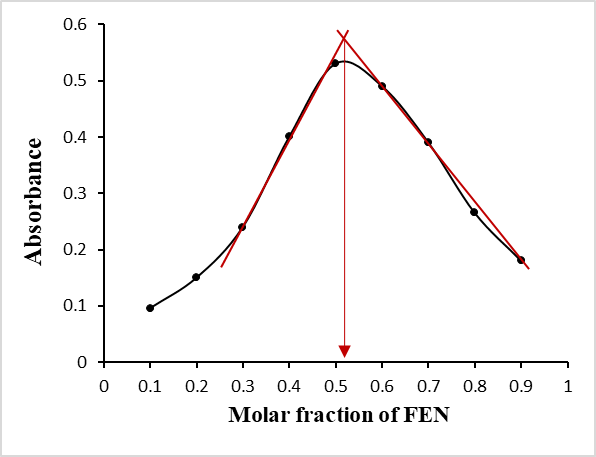


**(b)**

**Fig. S1.** Job’s continuous variation plots for determination of molar ratios of reaction between (a) ipratropium or (b) fenoterol and DDQ.


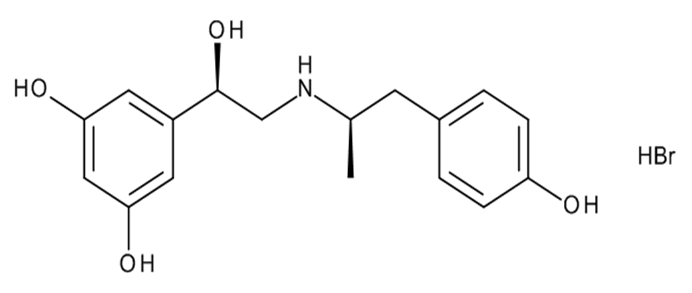


**(b)**


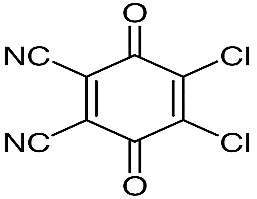

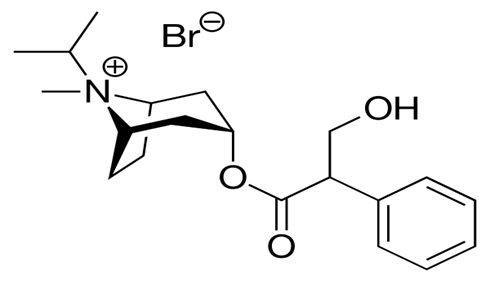


**(a)**


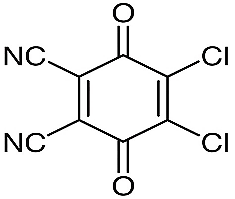


**Fig. S2.** Proposed reaction mechanism between charge transfer reagent; DDQ with (a) ipratropium bromide and (b) fenoterol hydrobromide.

**Table S1.** Comparative statistical analysis of the results obtained by the proposed colorimetric method and official methods for the analysis of ipratropium and fenoterol.

| **Parameter** | **Colorimetric method** | | **Official methods [21]** | |
| --- | --- | --- | --- | --- |
|  | **IPR** | **FEN** | **IPR** | **FEN** |
| **Mean** | 100.28 | 100.29 | 99.40 | 99.40 |
| **SD** | 1.832 | 1.701 | 0.926 | 1.062 |
| **n** | 6 | 6 | 6 | 6 |
| **Variance** | 3.348 | 2.890 | 0.857 | 1.128 |
| **Student’s t-test (2.228)^*^** | 1.050 | 1.087 | - | - |
| **F-test (5.05)^*^** | 3.907 | 2.562 | - | - |

^*^The values in parentheses represent the corresponding tabulated values of t and F at p=0.05.
